# An experimental multiplex PCR/RT-PCR for detection of the main viruses associated with equine neurological disease

**DOI:** 10.1007/s42770-026-02065-w

**Published:** 2026-08-18

**Authors:** S. O. Scherer, J. V. J. Silva Júnior, N. H. Pedroso, I. F. Dionisio, R. Weiblen, E. F. Flores

**Affiliations:** 1 Setor de Virologia, Departamento de Medicina Veterinária Preventiva, Universidade Federal de Santa Maria, Rio Grande do Sul, Brazil; 2 Programa de Pós-Graduação em Medicina Veterinária, Universidade Federal de Santa Maria, Rio Grande Do Sul, Brazil; 3 Departamento de Microbiologia e Parasitologia, Universidade Federal de Santa Maria, Rio Grande do Sul, Brazil; 4 Laboratório NB3 de Neuroimunologia, Universidade Federal de Santa Maria, Rio Grande do Sul, Brazil; 5 Programa de Pós-Graduação em Farmacologia, Universidade Federal de Santa Maria, Rio Grande do Sul, Brazil; 6 Setor de Virologia, Instituto Keizo Asami, Universidade Federal de Pernambuco, Pernambuco, Brazil

**Keywords:** Alphavirus, Equine herpesvirus, Flavivirus, Horse, Rabies

## Abstract

Viral neurological infections are important causes of morbidity and mortality in horses worldwide. Herein, we describe an end-point multiplex PCR/RT-PCR for simultaneous and differential detection of major equine encephalitis-related agents: rabies virus (RABV), equine herpesvirus type 1 (EHV-1), alphaviruses [Venezuelan equine encephalitis virus (VEEV), Eastern equine encephalitis virus (EEEV), Western equine encephalitis virus (WEEV) and Madariaga virus (MADV)] and flaviviruses [West Nile virus (WNV), Saint Louis encephalitis virus (SLEV), Rocio virus (ROCV) and Japanese encephalitis virus (JEV)]. The multiplex assay successfully amplified all targets either individually or simultaneously: alphavirus (381 bp—experimentally evaluated for MADV and potentially applicable to EEEV and WEEV based on primer design and sequence conservation), EHV-1 (255 bp), RABV (173 bp), VEEV (119 bp), and flavivirus (97-101 bp—experimentally evaluated using ROCV, with the potential to also detect WNV, SLEV and JEV, based on primer design and sequence conservation). Furthermore, the assay demonstrated analytical sensitivity and specificity that support its use for the comprehensive etiological diagnosis of equine neurological diseases. Overall, the multiplex PCR/RT-PCR described here may contribute to the diagnosis of viral neurological infections in horses, reducing costs and turnaround time, while contributing to prevention and control measures.

Neurological viral infections are amongst the main causes of morbidity and mortality in horses worldwide [1]. Viral agents involved in central nervous system (CNS) disease in horses include rabies virus (RABV), equine herpesvirus type 1 (EHV-1), flaviviruses [*e*.*g*., West Nile virus (WNV), Saint Louis encephalitis virus (SLEV), Rocio virus (ROCV) and Japanese encephalitis virus (JEV)] and alphaviruses [*e*.*g*., Venezuelan equine encephalitis virus (VEEV), Eastern equine encephalitis virus (EEEV) and Western equine encephalitis virus (WEEV) [2–6]. Furthermore, the alphavirus Madariaga virus (MADV) has been identified as an emerging equine and human pathogen in Latin America [7–9].

Neurological viral infections in horses frequently result in similar clinical signs [10], making accurate clinical diagnosis difficult. Thus, different laboratory strategies are available for identification of viruses involved in CNS disease, such as polymerase chain reaction (PCR) and reverse transcription-PCR (RT-PCR) [10, 11]. These assays allow the identification of encephalitis viruses; however, the diagnostic process may be time-consuming and/or expensive if each potential agent is investigated individually.

To contribute to the diagnosis of equine neurological infections, multiplex PCR and RT-PCR for simultaneous and differential identification of neurotropic viruses have been described [12–15]. Although these assays contribute to a faster diagnosis, they usually cover a limited number of targets. Furthermore, some of these approaches are based on real-time reactions, which are still unavailable in many diagnostic laboratories.

Herein, we describe an end-point multiplex PCR/RT-PCR for detection of four targets (potentially covering 10 viral species) associated with equine neurological disease: alphaviruses (EEEV, WEEV, VEEV and MADV), flaviviruses (WNV, SLEV, ROCV and JEV), EHV-1 and RABV, using primarily post-mortem encephalic (brain) samples. We believe this assay may contribute to the diagnosis of equine neurological infections, reducing its cost, time and helping with treatment/management, prevention and/or control measures.

Initially, we performed a detailed literature search in the PubMed and SciELO databases to identify high-coverage primers for all targets included in the multiplex assay. The putative amplicons were analyzed *in silico* for simultaneous identification and differentiation of all agents. Primers were considered suitable if they generated amplicons differing by at least 15 nucleotides (nt) in length from those of the other targets. As no primer sets meeting our criteria were identified, all primers used were manually designed in this study, without the use of primer design software, based on multiple sequence alignments of complete or near-complete genome sequences from GenBank, including 663 alphavirus, 43 EHV-1, 5 RABV (identified in horses from Brazil) and 798 flavivirus sequences. For alphaviruses, two primer sets were designed: one targeting EEEV, WEEV and MADV, and another specific for VEEV. Here, primer sets that anneal in all sequences in our database, with a maximum mismatch of 2 nt per primer for each virus, were considered. Furthermore, the primers were analyzed for non-specific annealing with other potential infectious agents and equine DNA/RNA (BLAST). Importantly, two and four adenine (A) nucleotides were added to the 5′ ends of the VEEV forward and reverse primers, respectively. These nucleotides are not part of the consensus viral sequences, but were intentionally incorporated to increase the amplicon size and facilitate its differentiation from the flavivirus amplicon. Since primer extension is primarily determined by the 3′ end, these 5′ additions are not expected to adversely affect primer annealing or amplification efficiency. Primer details are described in Table 1.

**Table 1:** Primers and targets used in the multiplex PCR/RT-PCR assay

Primer	Sequence (5′−3′)	Melting temperature (ºC)^b^	Genome	Virus	Target	Amplicon (bp)
Alpha E.W.M-F	GCAGGTCACYGACAATGACCATGC	58.4–59.7^c^	ssRNA^d^	EEEV, MADV, WEEV^f^	nsP1^k^	381
Alpha E.W.M-R1	GGTGCATGSACTGCRTAYACRTCYTGRTATAC	57.4–66.8^c^				
EHV1-F	GCTATAGTAGGCGGCGTTGT	56.8	dsDNA^e^	EHV-1^g^	ORF23^l^	255
EHV1-R	AGTGGTGCGCTTTTCGACTA	56.5				
RABV-F2	GAGGCTGAGATTGCTCACCA	58.6	ssRNA	RABV^h^	P^m^	173
RABV-R2	GATCCGTCATGGGCCAAACG	54.2				
VEEV-F3	^a^AAAGTTTGAGGTAGAAGCYAAGCAGGTCAC	54.3–56.4^c^	ssRNA	VEEV^i^	nsP1	119
VEEV-R3	AAAAGATCGTRTCGGATGGKTCYACCTCCGT	56.4–61.8^c^				
Flavi- J.R.W. S-F	AGGACTAGAGGTTAGAGGAGACC	54.7	ssRNA	JEV, SLEV, WNV, ROCV^j^	3UTR	97–101^n^
Flavi-J.R.W.S-R	GTCTTCCWCTAACCTCTAGTCCTTCC	66.4–64.7^c^				

^a^Underlined: non-consensus adenines added to facilitate differentiation of flavivirus amplicons

^b^Calculated at https://www.idtdna.com/calc/analyzer (for 50 mM monovalent salt, Na +), using primer concentrations optimized in the multiplex

^c^Temperature range due to degenerate nt

^d^single-stranded RNA

^e^double-stranded DNA

^f^Eastern equine encephalitis virus, Madariaga virus and Western equine encephalitis virus

^g^Equine herpesvirus type 1

^h^Rabies virus

^i^Venezuelan equine encephalitis virus

^j^Japanese encephalitis virus, Saint Louis encephalitis virus, West Nile virus and Rocio virus

^k^Nonstructural protein gene 1

^l^Open reading frame

^m^Phosphoprotein

^n^Range of base pairs due to nucleotide differences between target-viruses

Isolates/strains previously identified as MADV (XM6 164 strain) (provided by Dr. Laura Gil, Oswaldo Cruz Foundation, Aggeu Magalhães Institute, Brazil), VEEV (Dr. Luis Tadeu Figueiredo, University of São Paulo, Brazil), EHV-1 (Kentucky D strain; Dr. Rodrigo Franco, Butantan Institute, Brazil), RABV (CVS strain, Pasteur Institute, Brazil) and ROCV (Dr. Mara Elisa Gasino, Marcos Enrietti Diagnostic Center, Brazil) were used as positive controls for optimizing of the multiplex assay. *Streptococcus equi* subsp. *equi* (provided by Dr. Juliana Felipetto Cargnelutti, UFSM), *Trypanosoma evansi*, *Sarcocystis neurona* and *Neospora* spp. (Dr. Fernanda Silveira Flores, UFSM) and an EHV-4 isolate (Virology Section, SV-UFSM) were used as positive controls in the assessment of analytical specificity.

The multiplex assay was carried out with the AgPath-ID™ One-Step RT-PCR kit (Applied Biosystems™), optimizing the final concentrations of primers (0.2, 0.4 or 0.8 µM), enzyme (0.6 or 1 µL) and annealing/extension temperature (60, 62 or 64ºC). Multiplex PCR/RT-PCR products were stained with GelRed® (Biotium). The identity of amplicons obtained during optimization of the multiplex assay was confirmed by nucleotide sequencing.

The analytical specificity of the multiplex was analyzed against other potential neurological agents in horses: *S. equi*., *T*. *evansi*, *S. neurona, Neospora* spp. and EHV-4. The suitability of the DNA used in the assay was evaluated in reactions according to Cordoni et al. [16] (*S*. *equi*), Claes et al. [17] (*T*. *evansi*), Valadas et al. [18] (*S*. *neurona*), Müller et al. [19] (*Neospora* spp.), Lawrence et al. [20] (EHV-4). Potential non-specific amplifications of horse brain DNA/RNA were also analyzed. DNA/RNA extraction was performed according to Pedroso et al. [21].

The analytical sensitivity of the multiplex assay was determined using EHV-1, RABV, ROCV (representing the flavivirus group) and MADV (alphavirus group) strains. The LoDs were also determined in equine samples spiked with positive controls to assess whether the biological matrix could influence assay performance. Herein, RNA and DNA extraction was performed simultaneously using the PureLink™ Viral RNA/DNA Mini Kit (Invitrogen™).

Following the methodology above, the multiplex assay was optimized to 15μL final volume: 7.5μL of 2X RT-PCR buffer, 1μL of Taq Enzyme Mix, 0.24μL of forward and reverse primers (stock at 50 µM, final concentration 0.8 µM) for RABV and alphaviruses (EEEV, WEEV and MADV), 0.06 μL of forward and reverse primers for EHV-1, VEEV and flaviviruses (WNV, SLEV, ROCV and JEV) (stock at 50 µM, final concentration 0.2 µM) and 5.18μL of template (DNA and/or RNA). Reaction conditions were: 45 °C for 10 min (reverse transcription), 95 °C for 10 min (initial denaturation and reverse transcriptase inactivation), followed by 35 cycles of denaturation at 95 °C for 1 min, annealing/extension at 62 °C for 30 s. Multiplex amplicons were distinguished in 6% agarose gels (Fig. 1).

**Fig. 1:**
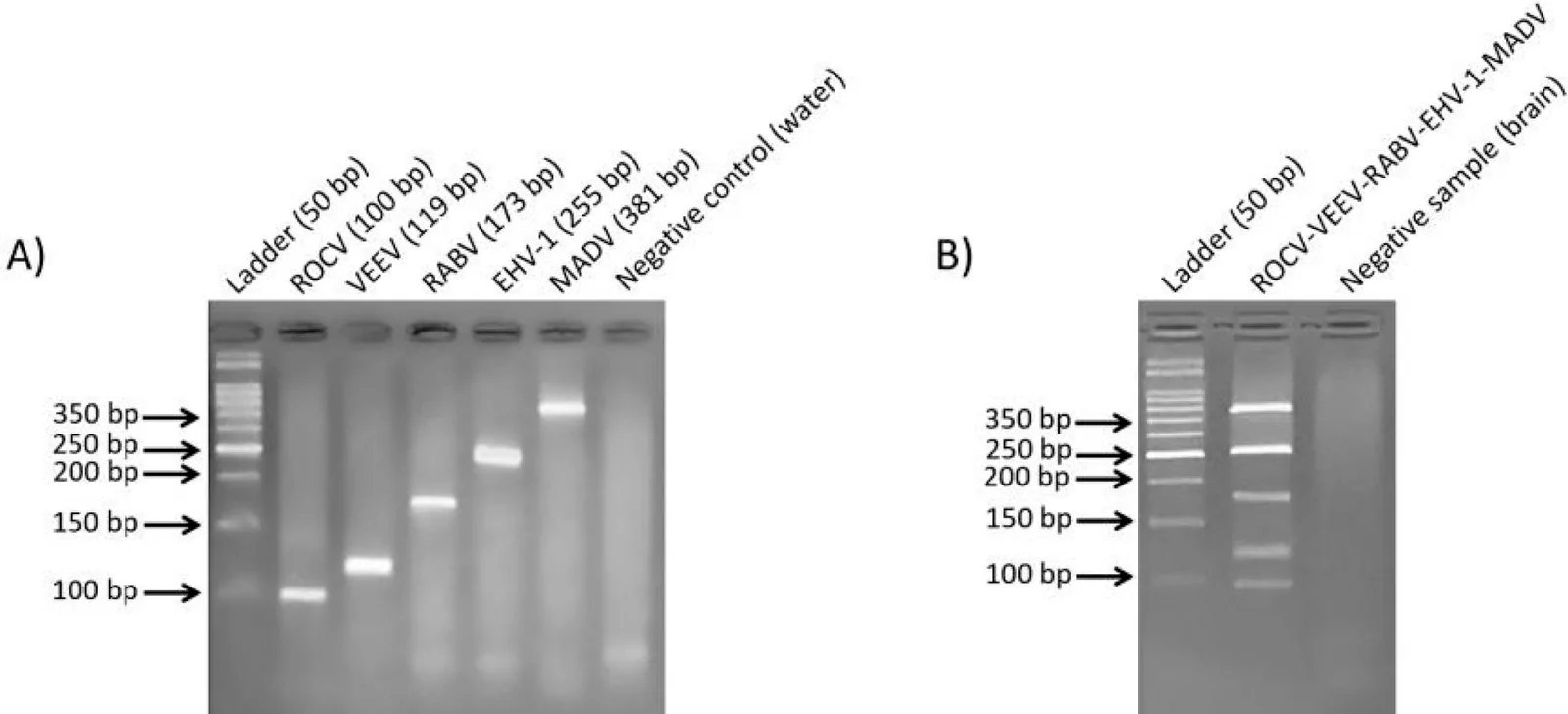
Performance of the multiplex PCR/RT-PCR. Individual (**A**) and simultaneous (**B**) amplification of the multiplex targets (positive controls). ROCV: Rocio virus; VEEV: Venezuelan equine encephalitis virus; RABV: rabies virus; EHV-1: equine herpesvirus type 1; MADV: Madariaga virus. In B, amplicons are in ascending order (ROCV > MADV)

The analytical specificity of the multiplex assay was tested with other agents potentially involved in equine neurological disease, as well as DNA/RNA from equine brain samples. No amplification was observed in any sample (Fig. 2). The LoD for RABV, EHV-1, ROCV and MADV was 1.6, 43.5, 50 and 53.7 TCID_50_, respectively (for both viral suspensions and spiked brain samples).

**Fig. 2:**
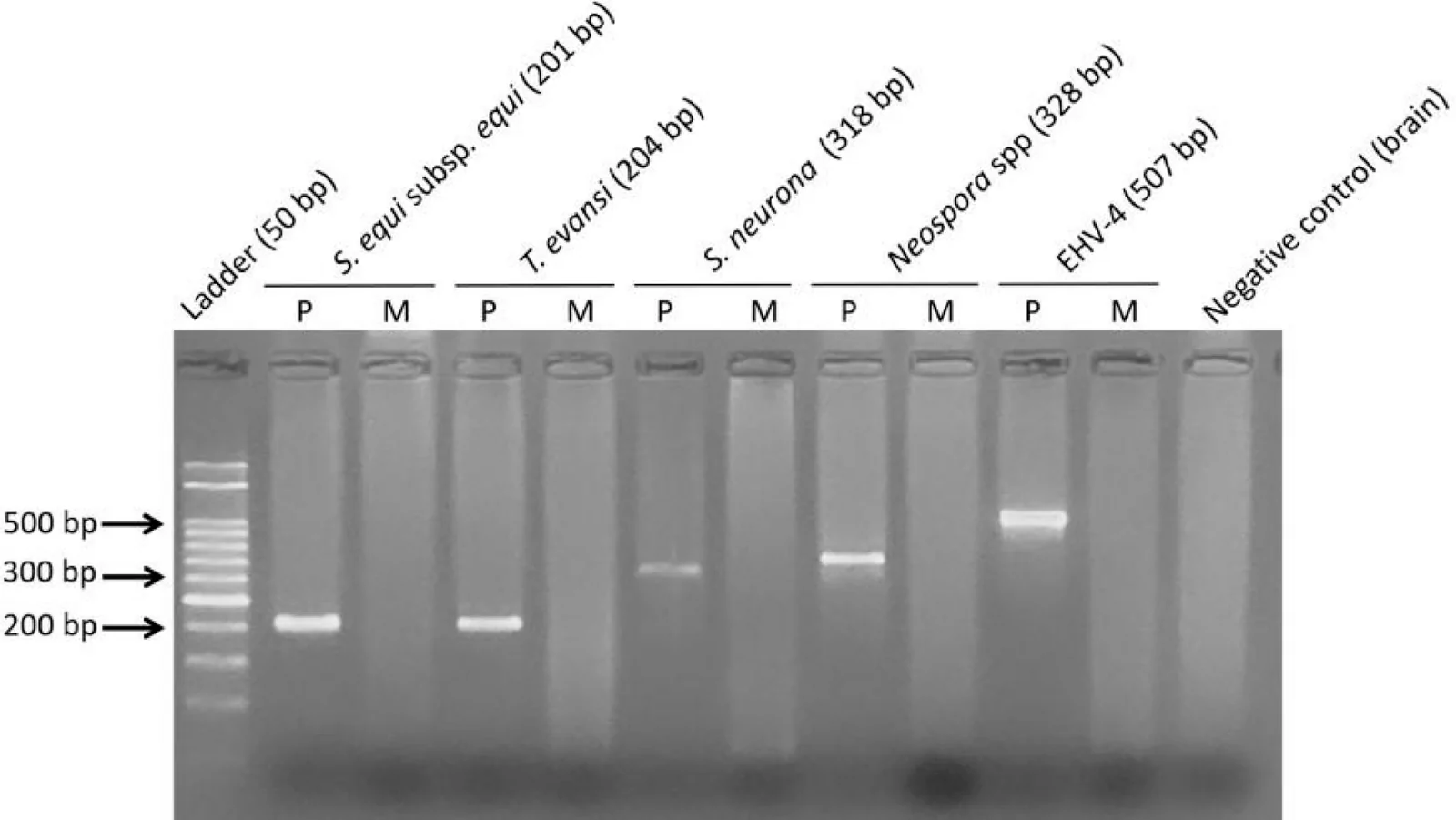
Analytical specificity of the multiplex assay. The analytical specificity of the multiplex was evaluated against *Streptococcus equi* subsp. *equi*, *Trypanosoma evansi*, *Sarcocystis neurona*, *Neospora* spp. and equine herpesvirus type 4. P: positive control performed with specific reactions for each target; M: no amplification in multiplex assay

Overall, we describe a multiplex PCR/RT-PCR for RABV, EHV-1, EEEV, WEEV, VEEV, MADV, WNV, SLEV, ROCV and JEV. Following a literature search for high-coverage primers that could be combined into an end-point multiplex, all primer sets used in the multiplex were designed in the study, using complete/near-complete genomes as reference. This strategy allowed us to identify conserved regions in the viral genomes, contributing to the high-coverage of the primer sets.

After primer design, the multiplex assay was standardized. The optimized multiplex assay was then able to simultaneously amplify all targets, regardless of the type of nucleic acid (DNA or RNA). The amplification of DNA and RNA targets in the same reaction represents an additional advantage of this assay, contributing to a faster and more accessible diagnosis. Furthermore, instead of performing protocols aimed at individual DNA or RNA extraction, we aligned the multiplex with simultaneous DNA/RNA extraction, using commercial kits or in-house protocols, which also accelerates the proposed strategy.

The requirement for size-distinguishable amplicons on agarose gels is a major limitation in end-point multiplex assays. Herein, we designed an assay that allows differentiation among five amplicons for detection of ten different viruses. Other assays for simultaneous detection of equine encephalitis agents have been described, however, they usuall cover only two or three viruses [12–14]. In addition, some of these assays are qPCR-based, which require specific reagents and equipment; or nested assays, which are time-consuming. In this perspective, our multiplex assay represents an alternative for laboratories lacking access to real-time platforms, in addition to allowing the detection of a higher number of viral agents. Importantly, due to the high risk of laboratory contamination in end-point PCR, particularly during post-amplification handling, appropriate precautions and good laboratory practices should be adopted to minimize the risk of contamination.

The multiplex assay developed in this study was also evaluated for non-specific amplification of other agents potentially involved in neurological disease in horses (EHV-4, *Neospora* spp., *T*. *evansi*, *S*. *neurona* and *S*. *equi*). Neither of the analyzed agents was amplified in the tests, confirming the specificity of the multiplex. The formation of primer dimers, which may be misinterpreted as amplicons, also represents a challenge in the end-point assay, especially in multiplex reactions with small targets. Here, primer dimers were occasionally observed, but did not influence the interpretation of the results, even in relation to the smallest amplicon (WNV-SLEV-ROCV-JEV, 97–101 bp).

Regarding analytical sensitivity, the LoDs for EHV-1, RABV, ROCV and MADV were determined. Although we did not have quantified isolates/strains of other flavivirus and alphavirus to evaluate the performance of our assay (EEEV, WEEV, VEEV, WNV, SLEV and JEV), the number of sequences and the criteria used for primer design, as well as the multiplex optimization process, support the suggestion that the multiplex would also adequately detect these targets. In addition, it is important to emphasize that the flavivirus and alphavirus primers were designed to detect members of their respective viral groups rather than individual virus species. Therefore, identification at species level cannot be achieved using the multiplex assay alone. In such cases, additional confirmatory approaches, such as species-specific PCR assays and/or sequencing of the amplified products, should be performed to accurately identify the infecting virus.

A limitation of the present study is the lack of suspicious/positive clinical samples for the multiplex targets. To circumvent this issue and to simulate routine detection, equine brain samples were spiked with the multiplex target viruses and used to evaluate the LoD in the same biological matrix used in the laboratory diagnosis of equine neurological diseases. Importantly, no influence of the sample type was observed on the multiplex performance, which maintained the same LoD observed in the test with viral suspensions from cell culture.

In conclusion, we describe an experimental end-point multiplex PCR/RT-PCR assay for detection of four targets, potentially covering up to ten viruses associated with viral neurological diseases in horses. This assay represents an accessible tool for investigation of equine neurological diseases, both for laboratory diagnosis and epidemiological studies.

## Data Availability

All data generated or analysed during this study are included in this published article.
