# Correction: An experimental multiplex PCR/RT-PCR for detection of the main viruses associated with equine neurological disease

**DOI:** 10.1007/s42770-026-02082-9

**Published:** 2026-09-03

**Authors:** S. O. Scherer, J. V. J. Silva Júnior, N. H. Pedroso, I. F. Dionisio, R. Weiblen, E. F. Flores

**Affiliations:** 1 Setor de Virologia, Departamento de Medicina Veterinária Preventiva, Universidade Federal de Santa Maria, Rio Grande do Sul, Brazil; 2 Programa de Pós-Graduação em Medicina Veterinária, Universidade Federal de Santa Maria, Rio Grande Do Sul, Brazil; 3 Departamento de Microbiologia e Parasitologia, Universidade Federal de Santa Maria, Rio Grande do Sul, Brazil; 4 Laboratório NB3 de Neuroimunologia, Universidade Federal de Santa Maria, Rio Grande do Sul, Brazil; 5 Programa de Pós-Graduação em Farmacologia, Universidade Federal de Santa Maria, Rio Grande do Sul, Brazil; 6 Setor de Virologia, Instituto Keizo Asami, Universidade Federal de Pernambuco, Pernambuco, Brazil

**Correction to: Brazilian Journal of Microbiology (2026) 57:241**

**https://doi.org/10.1007/s42770-026-02065-w**

In the original version of this article, the name of author S. O. Scherer has been incorrectly published as "S. O. de Scherer".

This error has been corrected in the original publication.

